# Patient Diagnostic Rate as Indicator of Tuberculosis Case Detection, South Africa

**DOI:** 10.3201/eid2203.151618

**Published:** 2016-03

**Authors:** Mareli M. Claassens, Cari van Schalkwyk, Rory Dunbar, Helen Ayles, Nulda Beyers

**Affiliations:** Desmond Tutu TB Centre and Department of Paediatrics and Child Health at Stellenbosch University, Tygerberg, South Africa (M.M. Claassens, R. Dunbar, N. Beyers);; The South African Department of Science and Technology/National Research Foundation Centre of Excellence in Epidemiological Modelling and Analysis at Stellenbosch University, Stellenbosch, South Africa (C. van Schalkwyk);; London School of Hygiene and Tropical Medicine, London, UK (H. Ayles); Zambart, Lusaka, Zambia (H. Ayles)

**Keywords:** Tuberculosis, HIV infections, South Africa, case detection, tuberculosis and other mycobacteria, bacteria, TB, HIV/AIDS and other retroviruses, viruses

## Abstract

To address the uncertainty of the indirectly measured tuberculosis case detection rate, we used survey data stratified by HIV status to calculate the patient diagnostic rate, a directly measurable indicator, in 8 communities in South Africa. Rates were lower among HIV-negative than HIV-positive persons. Tuberculosis programs should focus on HIV-negative persons.

The accuracy of the indirectly measured tuberculosis (TB) case detection rate is uncertain. A directly measurable indicator for TB case detection has been proposed (*1*) and subsequently used in analyses (*2*,*3*) from prevalence surveys. This indicator, the patient diagnostic rate (PDR), is defined as the rate at which prevalent case-patients are recruited by TB programs (*1*). It is estimated by dividing the notification rate (number of all newly notified cases/100,000 population/year) by the prevalence (number of new cases/100,000 population). To focus TB program efforts for case detection, PDR can be stratified by patient variables such as smear positivity, age, sex, and HIV status. To investigate differences in the rate at which cases are detected, we used data from the Zambia South Africa Tuberculosis and AIDS Reduction (ZAMSTAR) trial (*4*) prevalence survey. Before beginning the study, we obtained approval from the Health Research Ethics Committees of Stellenbosch University, the University of Zambia, and the London School of Hygiene and Tropical Medicine.

## The Study

The 2010 ZAMSTAR survey measured prevalence of culture-positive TB after 3 years of interventions in communities with a high TB/HIV burden. We selected communities that had a TB notification rate of >400 cases/100,000 population/year, were served by a healthcare facility offering TB diagnosis and treatment, and had a catchment area of >25,000 persons. Standard census enumerator areas were randomly selected within communities, and all adults (>18 years of age) from all households within the selected areas were asked to participate.

After obtaining written informed consent, we collected 1 sputum sample from each adult and offered HIV testing with 2 rapid HIV tests (Determine HIV-1/2, Alere, San Diego, CA, USA; and Uni-Gold, Trinity Biotech, Bray, Ireland). Participants who self-reported themselves as HIV positive were asked to be retested, but if they refused, they were not tested and were assumed to be HIV positive. Sputum samples were inoculated onto manual mycobacterial growth indicator tubes (Becton Dickinson, Franklin Lakes, NJ, USA), and identification of *Mycobacterium tuberculosis* isolates was confirmed by 16SrRNA sequencing (*4*).

Community notification rates were determined by using 2010 notification data from the electronic TB register of the community facility for the number of all newly notified cases (numerator) and 2011 census data for the estimated population size (denominator). To estimate the number of persons living with HIV, we stratified notification rates by using HIV data from the TB register for notified cases and by splitting the population per community into HIV positive or negative according to prevalence survey HIV results (Table 1). Prevalence rates were standardized by age and sex according to the 2011 census age/sex distribution per community. Prevalence data were stratified by HIV by using a survey variable that captured HIV test results combined with self-reported HIV status (Technical Appendix). The PDR per community, stratified by HIV status, was calculated by dividing notification rate by prevalence (Table 2).

**Table 1:** Tuberculosis notification rates per ZAMSTAR community in the Western Cape of South Africa, stratified by HIV status, 2010*

Community	Total†	Population, no. ‡			Notifications, no. (%)§				Notification rate¶	
		HIV–	HIV+		HIV–	HIV+	Missing HIV data		HIV–	HIV+
A	22,830	19,791	3,039		191	251	2 (0.5)		9.7	82.6
B	16,824	14,699	2,125		45	176	43 (16.3)		3.1	82.8
C	32,342	26,842	5,500		156	303	12 (2.6)		5.8	55.1
D	20,418	16,910	3,508		63	127	12 (4.9)		3.7	36.2
E	91,380	77,086	14,294		202	410	33 (5.1)		2.6	28.7
F	39,357	33,925	5,432		189	319	8 (1.6)		5.6	58.7
G	34,765	27,812	6,953		276	440	28 (3.8)		9.9	63.3
H	24,030	19,804	4,226		141	332	12 (2.5)		7.1	78.6
Total	281,946	236,869	45,077		1,263	2,358	150 (4.0)		5.3	52.3

*ZAMSTAR, Zambian South African Tuberculosis and AIDS Reduction trial; HIV–, HIV-negative; HIV+, HIV positive. †Split into HIV-positive and HIV-negative according to ZAMSTAR proportions. ‡Total adult (>18 years of age) population, data from 2011 census. §Data from 2010 electronic tuberculosis register. ¶Notification rate expressed per 1,000 persons per year.

**Table 2:** Patient diagnostic rate per ZAMSTAR community in the Western Cape of South Africa, stratified by HIV status, 2010*

Community	Patient diagnostic rate†	
	HIV– (95% CI)	HIV+ (95% CI)
A	0.74 (0.4–1.09)	1.63 (1.01–2.26)
B	0.30 (0.14–0.46)	4.16 (0.81–7.51)
C	0.28 (0.18–0.38)	1.61 (0.67–2.55)
D	0.27 (0.15–0.38)	0.81 (0.55–1.08)
E	0.23 (0.12–0.34)	1.09 (0.55–1.62)
F	0.33 (0.21–0.45)	2.01 (0.83–3.19)
G	0.61 (0.37–0.86)	2.64 (1.51–3.76)
H	0.35 (0.24–0.47)	1.99 (1.22–2.76)
Total	0.34 (0.29–0.39)	1.53 (1.27–1.79)

*ZAMSTAR, Zambian South African Tuberculosis and AIDS Reduction trial; HIV–, HIV-negative; HIV+, HIV positive. †Rate at which prevalent case-patients are recruited by tuberculosis programs, per person-year, calculated by dividing the notification rate by prevalence.

We assumed that the 2011 census would give an accurate estimate of the community population in 2010 and that the HIV prevalence in the community population would be similar to that in the survey population. We varied these assumptions according to estimated national population growth and national adult HIV prevalence in a sensitivity analysis; the effect was minimal (data not shown).

Overall, the PDR was 0.34 (95% CI 0.29–0.39) per person-year for the HIV-negative population and 1.53 (95% CI 1.27–1.79) per person-year for the HIV-positive population. In all 8 communities, the PDR was lower for the HIV-negative than the HIV-positive population (Table 2).

Study limitations included selection bias, which could have been introduced by sampling of areas with high notification rates. High notification rates could indicate a well-functioning reporting system, in contrast to areas with lower notification rates and possibly poorer reporting systems where similar prevalence rates could have been obtained. Similar prevalence rates with lower notification rates would have further decreased PDR. The uncertainty around HIV prevalence was not accounted for in the analysis. HIV status of survey participants combined self-reported data and HIV tests performed as part of the survey, but HIV status for a large number of participants remained unknown. Accurate data would have narrowed the 95% CIs. HIV results were sometimes missing from notification data, but unknown HIV status was not included in the analysis. HIV status information was missing specifically for 1 of the 8 communities (community B) and could have biased the results if a higher proportion of missing results were for HIV-positive and TB-negative persons, which would have meant a lower TB prevalence in the HIV-positive group and therefore a higher PDR. Few TB cases were diagnosed, leading to a small sample size, especially when the number of missing HIV results was considered. The assumption that the community size and age/sex distribution was the same in 2010 as in the 2011 census could have influenced the results.

## Conclusions 

In the absence of HIV infection, a PDR of >0.84 per person-year corresponds to the World Health Organization goal of detecting >70% incident cases according to the original Styblo model (*5*), assuming a disease duration of 2 years. However, when taking HIV status into account, disease durations of 3 years for HIV-negative and 0.93 years for HIV-positive persons are assumed (*6*), meaning that a case detection rate of >70% would correspond to a PDR of 0.78 among HIV-negative and 2.51 among HIV-positive persons. Our analysis showed that TB cases were detected at a lower rate among HIV-negative than among HIV-positive persons. None of the communities detected HIV-negative cases at a sufficient rate to limit transmission. Our results are specific to the ZAMSTAR communities in the Western Cape, which has many facilities with integrated TB/HIV programs and might not be representative of other non-ZAMSTAR settings, although TB/HIV integration is included in the South African National Tuberculosis Management Guidelines (http://www.sahivsoc.org/upload/documents/NTCP_Adult_TB%20Guidelines%2027.5.2014.pdf). However, our findings are similar to those of a study in Kenya (*2*), indicating that the PDR seems a consistent and appropriate statistic for evaluating case detection by using prevalence survey data from countries with a high burden of TB and HIV. A study of miners in South Africa showed that the duration of confirmed TB before diagnosis (calculated by dividing point prevalence by incidence) was 0.80 (95% CI 0.42–1.35) years among HIV-positive and 2.39 (95% CI 1.37–4.21) years among HIV-negative persons (*7*). These findings are similar to those of this study. Prevalence estimates were used in conjunction with incidence to determine disease duration; we used notification rates instead of incidence, in conjunction with prevalence estimates, to determine the rate at which TB programs recruit patients.

Case detection efforts should not focus on HIV-positive persons only, who seek healthcare earlier (*8*), are smear negative (*9*,*10*), and contribute less to community transmission (*10*); efforts should include strategies to detect HIV-negative patients who might contribute proportionally more to community transmission (*11*) and be accessing healthcare services already (*12*). HIV-negative persons who are more likely to be smear positive should undergo diagnostic testing for TB; for TB-positive persons, effective treatment should be started quickly (*13*). 

Our findings should be validated with analyses from other settings. Given the current World Health Organization focus on prevalence surveys, data for such analyses should be available. To help TB programs to develop active case-finding strategies, future research could investigate HIV-negative persons who are at risk of having TB and being missed by the healthcare system.

Technical Appendix Age/sex standardized tuberculosis prevalence in 8 communities in the Western Cape of South Africa, stratified by HIV status, 2010.
