# Supplementary material for: Patient Diagnostic Rate as Indicator of Tuberculosis Case Detection, South Africa
Source: Emerg Infect Dis. 2016 Mar;22(3):535–7. doi: 10.3201/eid2203.151618 (PMC4766915; doi:10.3201/eid2203.151618)
Supplement: Technical Appendix — Age/sex standardized tuberculosis prevalence in 8 communities in the Western Cape of South Africa, stratified by HIV status, 2010. [file 15-1618-Techapp-s1.pdf]

Article DOI: http://dx.doi.org/10.3201/eid2203.151618

Patient Diagnostic Rate as Indicator of Tuberculosis Case Detection, South Africa

Technical Appendix

Technical Appendix Table. Age/sex standardized tuberculosis prevalence per ZAMSTAR community in the Western Cape of South Africa, stratified by HIV status*

	Tuberculosis cases in ZAMSTAR, no.			Study sample in ZAMSTAR, no.			ZAMSTAR prevalence, cases/1,000		Standardized prevalence, cases/1,000†	
Community	HIV–	HIV+	Data missing	HIV–	HIV+	Data missing	HIV–	HIV+	HIV– (95% CI)‡	HIV+ (95% CI)‡
A	39	23	17	2,364	363	825	16.5	63.4	17.0 (10.83–26.64)	70.8 [48.58–101.96)
B	17	4	52	1,190	172	2,453	14.3	23.3	15.1 (9.41– 24.17)	28.6 (12.81–62.74)
C	49	18	41	1,918	393	1,564	25.5	45.8	26.1 (18.90– 35.99)	50.6 (28.15–89.46)
D	40	32	15	2,468	512	804	16.2	62.5	17.6 (11.93– 25.78)	62.9 (46.75–84.15)
E	25	14	17	1,936	359	1,467	12.9	39.0	14.0 (8.72– 22.31)	36.5 (22.42–58.90)
F	42	13	37	1,811	290	1,582	23.2	44.8	25.1 (17.76– 35.33)	45.7 (25.42–80.96)
G	39	19	33	2,000	500	1,308	19.5	38.0	19.9 (13.58– 29.04)	37.6 (24.63–57.06)
H	51	20	45	1,762	376	1,600	28.9	53.2	30.3 (22.53– 40.75)	59.7 (40.72– 86.76)
Total	302	143	257	15,449	2,965	11,603	19.5	48.2	20.5 (17.86– 23.61)	50.6 (42.92– 59.63)

*ZAMSTAR, Zambian South African Tuberculosis and AIDS Reduction trial; HIV–, HIV-negative; HIV+, HIV positive.
†Age/sex standardized according to 2011 census age/sex distribution for each community.
‡95% CIs adjusted for clustering at community level.
